# Extracellular vesicle-mediated drug delivery in breast cancer theranostics

**DOI:** 10.1007/s12672-024-01007-y

**Published:** 2024-05-23

**Authors:** Toufik Abdul-Rahman, Poulami Roy, Ranferi Eduardo Herrera-Calderón, Feriha Fatima Khidri, Quadri Ajibola Omotesho, Tolulope Sharon Rumide, Mahek Fatima, Sakshi Roy, Andrew Awuah Wireko, Oday Atallah, Subham Roy, Felix Amekpor, Shankhaneel Ghosh, Isaac Aksavdwa Agyigra, Viktoriia Horbas, Tetiana Teslyk, Valentyna Bumeister, Marios Papadakis, Athanasios Alexiou

**Affiliations:** 1 Medical Institute, Sumy State University, Sumy, Ukraine; 2 Department of Medicine, North Bengal Medical College and Hospital, Siliguri, India; 3 Center for Research in Health Sciences (CICSA), Faculty of Medicine, Anahuac University North Campus, 52786 Huixquilucan, Mexico; 4 Liaquat University of Medical and Health Sciences, Jamshoro, Pakistan; 5 Department of Biosciences and Nutrition, Karolinska Institutet, Stockholm, Sweden; 6 University of Ilorin, Ilorin, Nigeria; 7 Osmania Medical College, Hyderabad, India; 8 School of Medicine, Queens University Belfast, Northern Ireland, UK; 9 Department of Neurosurgery, Hannover Medical School, Carl-Neuberg-Strasse 1, 30625 Hannover, Germany; 10 Hull York Medical School, University of York, York, UK; 11 Noguchi Memorial Institute for Medical Research, University of Ghana, Accra, Ghana; 12 Institute of Medical Sciences and SUM Hospital, Siksha ‘O’ Anusandhan, Bhubaneswar, India; 13 Ahmadu Bello University, Zaria, Nigeria; 14 Department of Surgery II, University Hospital Witten-Herdecke, Heusnerstrasse 40, University of Witten-Herdecke, 42283 Wuppertal, Germany; 15 University Centre for Research and Development, Chandigarh University, Chandigarh-Ludhiana Highway, Mohali, Punjab India; 16 Department of Research and Development, Funogen, 11741 Athens, Greece; 17 Department of Research and Development, AFNP Med, 1030 Vienna, Austria; 18 Department of Science and Engineering, Novel Global Community Educational Foundation, Hebersham, NSW 2770 Australia

**Keywords:** Breast cancer, Theranostics, Extracellular vesicles

## Abstract

Breast cancer (BC) continues to be a significant global challenge due to drug resistance and severe side effects. The increasing prevalence is alarming, requiring new therapeutic approaches to address these challenges. At this point, Extracellular vesicles (EVs), specifically small endosome-released nanometer-sized EVs (SEVs) or exosomes, have been explored by literature as potential theranostics. Therefore, this review aims to highlight the therapeutic potential of exosomes in BC, focusing on their advantages in drug delivery and their ability to mitigate metastasis. Following the review, we identified exosomes' potential in combination therapies, serving as miRNA carriers and contributing to improved anti-tumor effects. This is evident in clinical trials investigating exosomes in BC, which have shown their ability to boost chemotherapy efficacy by delivering drugs like paclitaxel (PTX) and doxorubicin (DOX). However, the translation of EVs into BC therapy is hindered by various challenges. These challenges include the heterogeneity of EVs, the selection of the appropriate parent cell, the loading procedures, and determining the optimal administration routes. Despite the promising therapeutic potential of EVs, these obstacles must be addressed to realize their benefits in BC treatment.

## Introduction

Breast cancer (BC) stands as the most frequently diagnosed cancer globally, representing the primary cause of cancer-related mortality in females. In 2020, BC comprised 11.7% of newly reported cancer cases worldwide, totaling 2.26 million instances and contributing to 6.9% of cancer-related deaths [1, 2]. Incidence exhibits a robust correlation with human development, manifesting higher rates in developed countries [2]. However, less developed nations experience more elevated mortality rates, primarily due to late detection and limited access to diverse treatment modalities [3, 4]. In regions like South America, Africa, and Asia, there is a notable upward trend in BC incidence, potentially stemming from lifestyle changes and expanded screening programs [3]. Approximately 10% of BC cases are hereditary, with lifestyle factors contributing to overall risk [5, 6].

Early diagnosis assumes paramount importance, given the substantial disparities between early-stage (96% 5-year survival) and metastatic BC (38% 5-year survival) with profound implications for prognosis [7]. The diagnostic process involves mammography screening, a proven method that reduces BC mortality by 19% [8]. For higher-risk patients, supplementing mammography with Magnetic Resonance Imaging (MRI) is recommended, enhancing the detection of occult cancers [9, 10]. However, early diagnosis remains challenging, necessitating the development of more effective methods.

BC exhibits molecular heterogeneity, presenting subtypes such as luminal A, luminal B, basal-like, and Human epidermal growth factor receptor 2 (HER2)-enriched [11]. Treatment strategies take this diversity into account, emphasizing biologically-directed therapies and treatment de-escalation. Available treatment modalities encompass surgery, radiation, chemotherapy, and hormonal therapy. Despite notable advancements, the recurrence and metastasis of BC persist, often attributed to therapy-resistant cells, serving as predominant causes of death [6, 12–14]. Metastases alone account for over 90% of BC-related fatalities [15]. Consequently, addressing the determinants of distant metastasis and therapy resistance is essential for devising more effective therapeutic strategies.

Over the years, cancer management has relied on methods such as imaging, chemotherapy, radiotherapy, and surgery. However, these approaches face challenges such as incomplete resection, off-target toxicities, and limited drug penetration into tumors [16, 17]. The emergence of personalized medicine, especially in theranostics, addresses these issues by tailoring treatments to patient needs [18]. Theranostics is a term coined in 1998 by John Funkhouser, and it refers to an approach combining therapy and diagnostics for disease diagnosis, treatment, and follow-up [19]. Nevertheless, the history of radiotheranostics dates back to 1941 when Saul Hertz pioneered the use of radioiodine for thyrotoxicosis treatment [20]. Since then, the integration of diagnosis and therapy has become common, with recent advancements targeting somatostatin receptors in neuroendocrine tumors, HER2 antigens in BC, and Prostate-specific membrane antigen (PSMA) in prostate cancer [20–22]. Nanotechnology, specifically Extracellular Vesicles (EVs) such as exosomes, explores targeted therapy and biomarker identification in BC, offering a non-invasive alternative for early cancer detection [23]. It has been demonstrated that exosomes, nanovesicles facilitating cellular communication, offer a non-invasive alternative for clinical applications, addressing challenges in early cancer detection posed by tumor heterogeneity and conventional biopsy methods [23, 24]. Theranostics holds promise for effective and safe BC therapy, combining cutting-edge technologies into a single platform for personalized medicine.

Over the past decade, the field of EVs has experienced significant growth, showcasing diagnostic, prognostic, and therapeutic potential [25]. They are present in biological fluids such as saliva, urine, milk, and amniotic fluid and are classified as exosomes, microvesicles, and apoptotic bodies [26–28]. EVs play a crucial role in cell communication by carrying nucleic acids and specific proteins/lipids [29]. In the context of BC, EVs are implicated in tumor microenvironment (TME) modulation, angiogenesis, metastasis, and drug resistance [30–32]. It has been demonstrated that EVs have a significant impact on cancer progression, and they also contribute to drug resistance, posing challenges in anti-cancer treatments [30, 33]. However, there is growing optimism as EVs show potential as biomarkers. As mentioned above, early diagnosis of BC is crucial for survival prognosis, but despite the fact that we already have some cancer biomarkers such as tissue receptor expression (Estrogen Receptor (ER), Progesterone Receptor (PR), HER2) that are vital for staging or blood biomarkers, like Cancer Antigen (CA) 15-3, CA27-29, and Carcinoembryonic Antigen (CEA), these have limited sensitivity in early BC [34]. EVs could offer a less invasive and more effective alternative, even serving as a tool for monitoring disease progression and treatment efficacy [35, 36]. For example, it has been proposed to use circulating exosomal micro-ribonucleic acids (miRNAs), long noncoding Ribonucleic Acids (lncRNAs), and proteins as potential diagnostic tools for BC [37]. Moreover, due to their biocompatibility that allows them to cross biological barriers such as the blood–brain barrier, EVs could be used as natural drug delivery vehicles, overcoming limitations associated with conventional treatments; such as toxicities associated with cell-based therapies, they can carry proteins, miRNAs, siRNAs, and other therapeutic compounds [29, 38–43]. This could improve drug penetrance, stability, and cellular uptake in targeted sites [44].

This article seeks to delve deeply into the multifaceted potential of EVs as both biomarkers and vehicles for drug delivery. Our goal is to offer a comprehensive analysis of the mechanisms, diverse applications, and challenges inherent in harnessing the capabilities of EVs in these roles.

## Methodology

A contemporary and comprehensive narrative review on "Extracellular Vesicle-Mediated Drug Delivery in Breast Cancer Theranostics" was conducted, utilizing PubMed, SCOPUS, and Google Scholar as the primary databases. Precise keywords and MeSH terms, including "extracellular vesicles," "drug delivery," "breast cancer," and "theranostics." were employed to identify relevant articles for the study. Furthermore, a manual search was conducted to identify references from recently published studies. The inclusion criteria encompassed English-language articles, comprising research studies and clinical investigations directly addressing the use of extracellular vesicles in breast cancer theranostics. No specific time frame was set for study inclusion, however, priority was given to recently published studies to ensure recent advances on the topic. All unpublished articles were excluded from this study. Furthermore, articles were excluded if they did not align with the primary focus of the study or lacked sufficient information for analysis.

## EVs

**Fig. 1:**
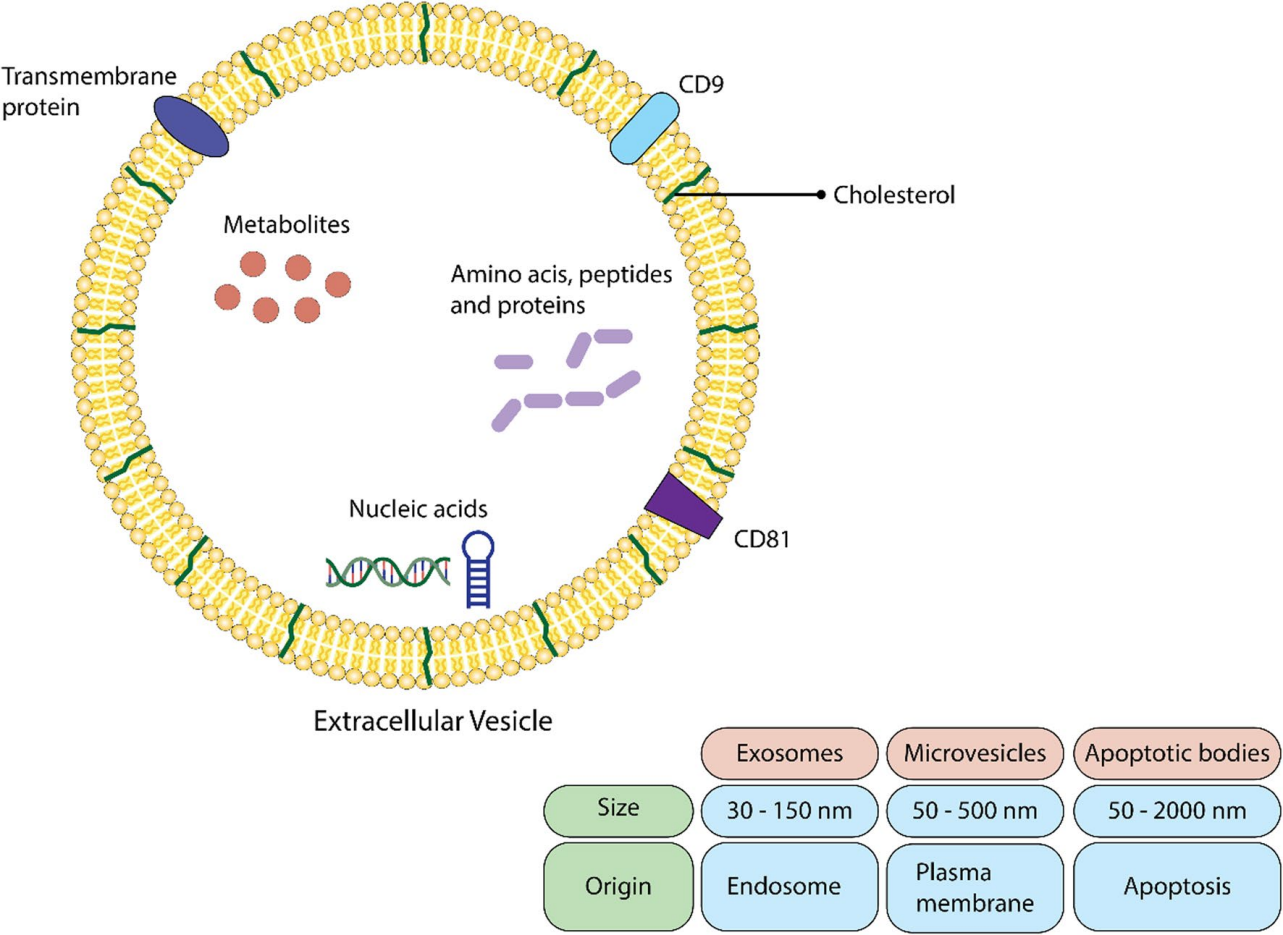
Structure and Types of Extracellular Vesicles. Extracellular vesicles (EVs) are lipid-bound structures secreted by cells into the extracellular space. They encompass microvesicles, exosomes, and apoptotic bodies, each distinguished by their origin, size, and cargo, comprising lipids, nucleic acids, and proteins from various cellular compartments

EVs are small, lipid-bilayer membrane-derived particles released from cells into the extracellular space (Fig. 1) [45]. First reported in 1946 by Erwin Chargaff and Randolph West, EVs have garnered increasing interest due to their involvement in various physiological conditions, including cancer [45, 46]. They serve as messengers, transporting proteins, nucleic acids, lipids, and other molecules between cells and their microenvironments [47, 48]. The International Society for Extracellular Vesicles (ISEV) recommends classifying EVs based on physical characteristics [28]. Exosomes, microvesicles, and apoptotic bodies are categorized by size and generation mechanism, influencing their uptake and cargo fate.

The 2018 Minimal Information for Studies of Extracellular Vesicles (MISEV) consensus recommends distinguishing exosomes or exosomes and medium/large extracellular vesicles (M/LEVs) based on size [28]. exosomes undergo endosomal cargo sorting, leading to intraluminal vesicles within multivesicular bodies, while M/LEVs originate from outward plasma membrane budding [49, 50]. Despite distinct biogenesis mechanisms, both exosomes and M/LEVs may function similarly in intercellular communication within the TME [51].

In the BC-TME, EVs play a crucial role in mediating cell signaling [51]. These EVs transport oncogenic proteins, lipids, miRNA, and DNA, influencing signaling and gene regulation [52]. Protein cargo involves molecules in signal transduction and immunoregulatory molecules [53]. Nucleic acid cargo varies among EV subtypes, with miRNAs being potent regulators of gene expression [54, 55]. Less is known about lipid cargo, but sphingomyelin, glycosphingolipids, and phosphatidylserine are enriched in EVs [56]. Furthermore, EV uptake leads to "cancer-induced reprogramming," reinforcing cancer progression and contributing to the creation of favorable environments for metastasis [57–61].

## EVs in BC progression

The liberation of exosomes from tumor cells represents a pivotal mechanism governing intercellular communication within the TME, exerting profound influence over key cancer hallmarks [62]. Initially discovered several decades ago, the significance of exosomes was markedly underscored in 2007 with the revelation of their Ribonucleic acid (RNA) content, encapsulated within these nanoscale vesicles [63]. In the context of BC, a condition characterized by a spectrum of clinicopathological features, the dynamic interplay between cancerous cells and the non-malignant microenvironment assumes paramount importance [64–67]. This microenvironment comprises diverse cell types, encompassing cancer-associated fibroblasts (CAFs), immune cells, and extracellular matrix (ECM) components 68], [69]. Exosomes, functioning as positive modulators, assume a central role in guiding BC through its phases of development, progression, invasion, metastasis, stimulation of stem cells, and resistance to therapeutic interventions [70–72]. The intricate nature of these interactions is accentuated by the tissue-specific inhibition of exosome release from BC cells, mediated by exosomes derived from normal mammary epithelial cells [73].

### TME

The intricate interplay between the TME and exosomes assumes a pivotal role in sculpting the dynamics of cancer progression, particularly in breast tissue. Under stress-induced conditions, the liberation of exosomes from tumor cells serves as a trigger for TME changes and expansion [74]. These exosomes, laden with bioactive molecules, function as messengers, intricately coordinating the modulation of gene expression within the TME [53, 55]. In the context of breast tissue, exosome-mediated delivery of miRNAs exercises a significant influence over cytokines and growth factors, consequently impacting the ECM and contributing to the underlying pathogenesis [75].

​​The complexities of this communication network extend beyond the confines of cancer cells. exosomes, as mediators, facilitate interactions between mesenchymal stem cells (MSCs) and cancer cells, thereby fostering processes such as angiogenesis, invasion, drug resistance, and the establishment of dormant micrometastasis, or reversal of dormancy [68, 76–78]. Moreover, exosomes originating from both fibroblasts and CAFs induce phenotypic shifts in BC cells, cultivating chemoresistance and enabling anchorage-independent growth [79, 80]. This multifaceted interplay underscores the complexity of the molecular landscape governing BC progression.

### Tumor proliferation

The proliferation of tumors in BC is a complex process driven by intricate interactions facilitated by exosomes released by specific BC cells. These exosomes play a significant role in tumor expansion and oncogenic potential by encapsulating lncRNAs that function as oncogenes, thereby promoting tumor growth through the upregulation of critical signaling pathways [81]. Importantly, oncoproteins such as Myelocytomatosis (MYC) and Aurora Kinase B (AURKB) intricately regulate the biogenesis and release of these exosomes. Tumorigenesis and proliferation are further propelled by diverse functional cargos delivered via exosomes. Noteworthy examples include Nischarin-positive exosomes, which promote BC cell migration, and miR-1246, which regulates cell cycle progression. Moreover, exosomes containing the metastasis-associated lung adenocarcinoma transcript 1 (MALAT1) contribute to an increase in tumor size, while the programmed death-ligand 1 (PD-L1) in exosomes suppress T cell activity, thereby fostering tumor proliferation [82, 83].

The influence of exosomes extends beyond cancer cells to fibroblast and stromal activation. Exosomes derived from BC cells have the capacity to transform normal fibroblasts into CAFs, thereby contributing to the aggressive nature of the disease [84, 85]. Within the TME, adipocytes, known as cancer-associated adipocytes (CAAs), play a pivotal role in promoting BC development through various signaling pathways, including the fibroblast growth factor (FGF), the vascular endothelial growth factor (VEGF), and Interleukin-1 beta ( IL-1β) [86]. In addition, adipose tissue-derived MSCs induced by BC-derived EVs undergo differentiation into tumor-associated myofibroblasts, promoting both migration and proliferation [87].

Furthermore, the uptake of EVs from MDA-MB-231 cells by MCF10A cells induces regulatory changes in E-cadherin, secretion of matrix metalloproteinases (MMPs), and promotion of invasion [88]. Additionally, the reprogramming of cancer metabolism, an essential process for cancer progression, is facilitated by exosomes shed from BC cells and the TME, carrying metabolites and enzymes that affect glycolysis and other critical pathways [37]. For example, exosomes from MDA-MB-231 BC cell line enhance the expression of Glucose Transporter 1 (GLUT1) and hexokinase HK2 genes in peripheral blood mononuclear cells, promoting glycolysis and cell proliferation [89].

### Angiogenesis

In the context of BC, the initiation of angiogenesis is facilitated by the action of exosomes. Under conditions of reduced oxygen availability, an upregulation in exosome secretion ensues, thereby promoting the formation of new blood vessels, a pivotal process that underpins tumor proliferation and metastasis [90]. Notably, it has been reported that hypoxic tumors release exosomes that possess an augmented capacity to instigate angiogenesis and vascular permeability, a phenomenon mediated through the activation of hypoxia-inducible factor-1 alpha (HIF-1α) signaling [91]. Additionally, within these exosomes, miRNAs such as miR-9 and miR-23a play a significant role in inducing endothelial angiogenesis by modulating specific signaling pathways [92, 93].

Furthermore, exosomes originating from BC cells, in tandem with the secretion of transforming growth factor-beta (TGF-β) and VEGF, contribute to the myofibroblastic differentiation of adipose-derived stem cells (ASCs), activate Mitogen-activated Protein kinase (MAPK) signaling pathways in ASCs and promote ASC pro-angiogenic behavior [94]. Moreover, serum exosomes annexin A2 has emerged as a key player directly implicated in angiogenesis [95, 96]. Its levels exhibit a robust correlation with tumor grade and significantly impact overall and disease-free survival in the context of triple-negative breast cancer (TNBC) [96].

### Immune evasion

Immune evasion mechanisms in BC encompass intricate interactions facilitated by exosomes, which play a pivotal role in the crosstalk between cancer cells and immune cells. Particularly, tumor-derived exosomes carrying surface markers such as PD-L1 and miR-92 have been identified as key mediators delivering negative signals to immune cells [83, 97–99]. This phenomenon promotes immunosuppressive effects by inducing T-cell exhaustion and suppressing Natural Killer (NK) cell cytotoxicity [100]. Furthermore, the secretion of exosomes by BC cells, induced by hypoxia, contributes to T-cell suppression through the action of TGF-β [101].

BC cell-derived exosomes additionally contribute to immune evasion by engaging with various immune cells, including dendritic cells (DCs), macrophages, and T-regulatory cells. This interaction fosters cancer progression and facilitates distant metastasis. Tumor-associated macrophages (TAMs) adopt an immunosuppressive M2 phenotype through EV-mediated communication with cancer cells [102–104]. On the other hand, DCs have been shown to employ EVs for anti-tumor immune responses, however, BC-derived vesicles exhibit the capacity to alter fatty acid metabolism in DCs, enhancing immune evasion [105, 106]. It has been demonstrated that myeloid-derived suppressor cell (MDSC)-derived vesicles are key mediators in promoting growth, invasion, and angiogenesis in BC [107, 108]. Moreover, it has been shown that murine BC cell-derived exosomes induce the accumulation of MDSCs in the lungs and liver, suppressing NK cell cytotoxicity and ultimately conditioning the pre-metastatic niche [109]. Furthermore, although research is limited, some studies have reported that BC-derived vesicles influence neutrophil levels and thrombus formation, potentially impacting disease progression [110].

### Metastasis

Invasion and migration, pivotal elements in the genesis of treatment resistance, are facilitated by BC-derived exosomes that encapsulate metalloproteinases responsible for the degradation of the ECM [111]. exosomes originating from BC cells play a sophisticated role in ECM remodeling, promoting invasiveness, and enabling the local dissemination of tumor cells, often accompanied by vascular disruption [112–115]. Extending beyond the confines of the primary tumor, these exosomes actively shape a pre-metastatic niche, steering distant sites toward a hospitable environment conducive to metastatic settlement [57, 116]. Notably, the miR-200 family, encapsulated within exosomes from BC cells, emerges as a potent mediator of metastatic signals conveyed to distant tumor cells [117].

In the context of brain metastases in BC, exosome-derived miR-1290 assumes significance, activating astrocytes and propelling metastasis within the brain [118]. Parallel findings indicate that exosomes from metastatic BC cells in the brain carry miR-181c, facilitating blood–brain barrier destruction and mediating brain metastasis [119]. Additionally, Integrin avb1, conveyed by circulating exosomes, establishes a molecular link with metastatic BC cells, suggesting its potential involvement in the metastatic cascade [120]. Furthermore, miR-105 from BC-associated EVs strategically suppresses Zonula occludens-1 (ZO-1) expression, leading to the dismantling of cell–cell adhesion and fostering metastasis [114]. Moreover, exosomes rich in miR-122, emanating from BC cells, ingeniously reprogram glucose metabolism in pre-metastatic niches, thereby propelling the machinery of metastasis [121]. In addition, exosomes derived from MSCs in the bone marrow induce dormancy in metastatic BC cells, contributing significantly to the prolonged latency periods observed in metastatic disease [122].

### Drug resistance

Tumor-derived exosomes have emerged as pivotal entities in the intricate landscape of drug resistance within the context of cancer. These vesicles play a crucial role by facilitating the transfer of functional resistance proteins between cancer cells, thereby orchestrating pathways that actively promote chemotherapeutic drug efflux [123]. Exosomes are known to mediate at least three pathways that promote chemotherapeutic drug efflux. They contribute to drug efflux directly, enhance the expression and function of membrane-embedded drug efflux pumps, and influence the expression of proteins and miRNAs that regulate specific drug efflux proteins, such as P-glycoprotein (P-gp) [124–128].

In BC research, early investigations into drug resistance focused on intracellular vesicles associated with resistance to mitoxantrone in the MCF-7 cell line. These vesicles contained the ABCG2 protein, contributing to drug resistance [129]. Now, the significance of exosomes in the realm of drug resistance is underscored by specific miRNAs identified within their cargo. Notably, miR-9-5p and miR-101 have been implicated in tamoxifen resistance, exerting their influence by downregulating target genes [130, 131]. Furthermore, exosomes from tamoxifen-resistant variants were found to transfer miR-221/222 [132]. Similarly, miR-21 present in exosomes has been linked to trastuzumab resistance, further accentuating the multifaceted role of these vesicles in conferring resistance [133, 134]. Moreover, it has been shown that exosomes from adriamycin- and docetaxel-resistant cell lines transfer resistance to drug-sensitive cells, involving specific miRNAs (miR-100, miR-222, miR-30a, and miR-17) [135–137]. Expanding the scope, exosomes-carried lncRNAs have also been implicated in promoting drug resistance in BC [138]**.**

## Theranostic application of EVs as biomarkers for BC diagnosis

In recent decades, scholars have delved into the examination of EVs in the context of BC. Research findings underscore the substantial engagement of EVs in key pathways pivotal to the development of BC, encompassing processes like proliferation, migration, modulation of the TME, and the emergence of drug resistance [139, 140]. Furthermore, a multitude of clinical investigations have underscored the prospective utility of EVs in both therapeutic interventions and diagnostic frameworks for BC [33, 141]. Exosomes encapsulate an extensive repertoire, approximately half, of the human proteome [142]. This diverse protein content, mirroring the cell types of origin, positions exosomes as an ideal candidate for discovering disease-specific biomarkers, particularly in the context of BC (Table 1 [96, 111, 114, 143, 144]).

**Table 1:** Exosome Biomarkers Detection in Breast Cancer

Study/Reference	Biomarker name	Source of biomarker	Detection method(s)	Clinical relevance
Zhou et al*.* [114]	miR-105	Patient solid and liquird tumor biopsies	Immunohistochemistry	Detection of exosomal miR-105 in early breast cancer patient biopsies indicates metastatic progression
Bandini et al*.* [143]	Exosomal surface epitopes and Isotopes	BC patient plasma and cell line supernatants	Flow Cytometry	The expression of EV-related biomarkers in BC patient plasma and cell line supernatants might be used to characterise and track disease development
Khan et al [111]	Exosome protein survivin-2B	Serum exosome	Western blots and immunohistochemistry	In early breast cancer patients, differential expression of exosomal-Survivin is a prognostic or diagnostic marker
Li et al*.* [144]	Exosomal miR-148a	Serum exosome	Quantitative Real Time-Reverse Transcription Polymerase Chain Reaction (qRT-PCR)	Decreased levels of serum exosomal miR-148a is strongly related with a poor clinical outcome of BC, implying that serum exosomal miR-148a could serve as a viable diagnostic and prognostic biomarker for BC
Chaudhary et al*.* [96]	Serum exo-AnxA2	Serum exosome	In vivo Matrigel plug assay	Exo-AnxA2 is a possible prognosticator of triple-negative breast cancer, which might lead to an efficient therapeutic alternative

Axillary lymph node (ALN) metastasis stands out as a crucial prognostic factor in early-stage BC [145]. Sentinel lymph node biopsy (SLNB) is a primary method for assessing ALN status, but its limitations, including a notable false-negative rate and postoperative complications like lymphedema, necessitate alternative approaches [146].

Numerous studies emphasize the pivotal role of exosome protein biomarkers in BC diagnosis. Lee et al., through Liquid Chromatography-Tandem Mass Spectrometry (LC–MS/MS), identified 270 exosome proteins in invasive BC cell lines, unveiling the diagnostic biomarker Epidermal Growth Factor-like repeats and Discoidin I-Like Domains 3 (EDIL3) correlated with metastasis [147]. In another investigation, the profiling of 241 uniquely expressed exosome proteins in various BC cell lines pinpointed fibronectin (FN) as a promising diagnostic biomarker, specifically distinguishing between ER + and ER − BC [36, 148].

Wang et al.'s study introduced miR-363-5p in plasma exosomes as a diagnostic indicator for distinguishing ALN-positive and ALN-negative BC. Moreover, high expression of miR-363-5p in plasma exosomes correlates with prolonged survival, highlighting its potential as a diagnostic marker with prognostic value [149]. The expression of Connexin-46 (Cx46) in EVs released from BC cells has garnered significant attention. Cx46 plays a pivotal role in enhancing the interactions between EVs and receptor cells, thereby contributing to the migratory and invasive abilities of BC cells. This identification positions EVs-Cx46 not only as a potential malignancy marker for BC but also as a viable target for therapeutic interventions [150]***.***

**Fig. 2:**
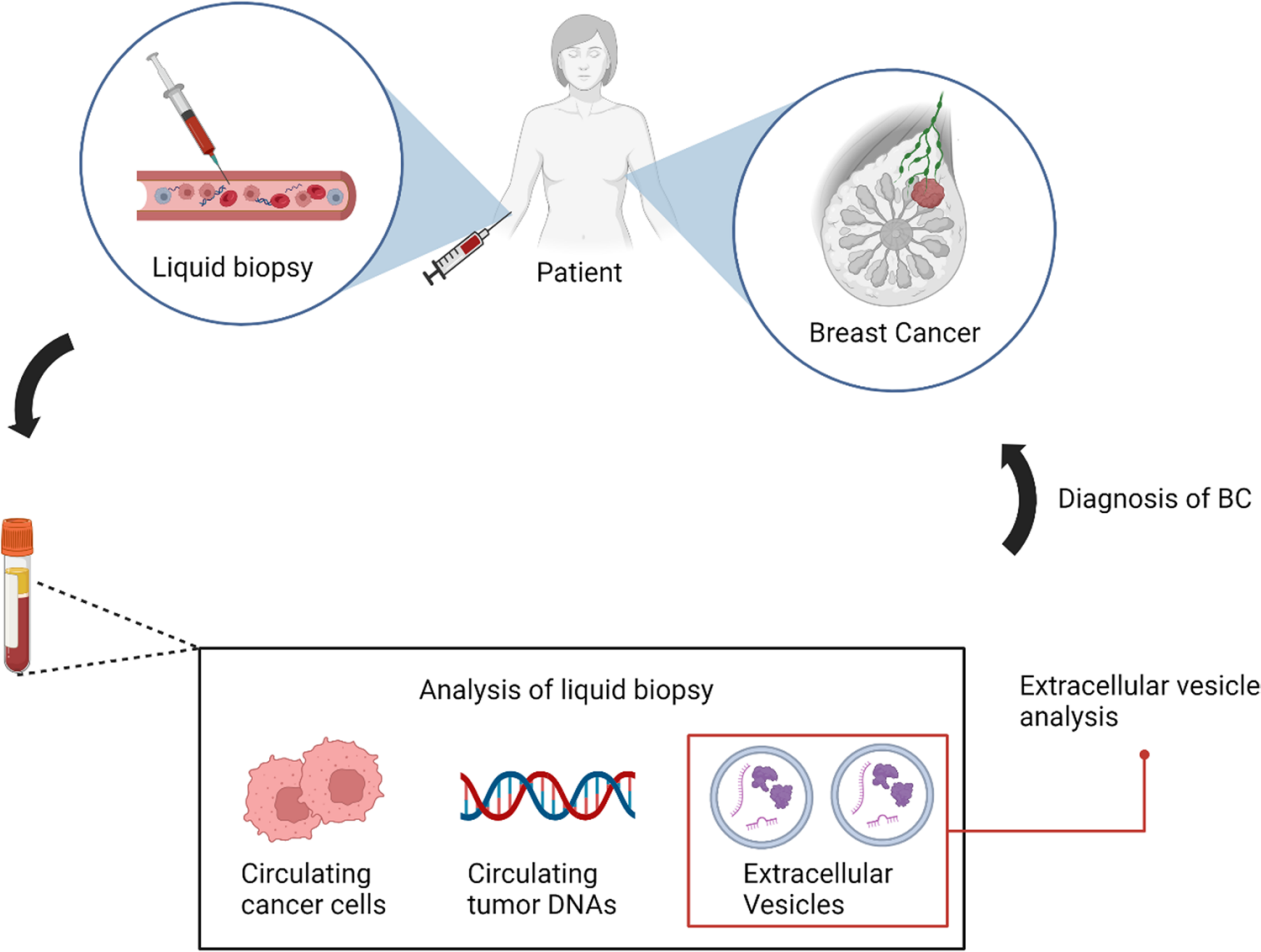
Liquid Biopsy in Breast Cancer. Liquid biopsy in breast cancer reveals miRNA profiles in exosomes, aiding in recurrence risk assessment and early detection strategies. Exosome-derived miRNA profiling distinguishes breast cancer recurrence risks

The concept of liquid biopsy (Fig. 2) for studying recurrence risk and early detection in tumor patients takes a spotlight, specifically focusing on multiple miRNAs contained in BC cell-secreted exosomes [67, 151–153]. The utilization of exosome-based liquid biopsy emerges as a crucial element for refining treatment decisions and prognostic evaluation. A comprehensive exosome miRNA profiling study brought to light the prognostic potential of specific miRNAs in BC recurrence. Elevated levels of miR-338-3p, miR-340-5p, and miR-124-3p were identified, contrasting with the down-regulation of miR-29b-3p, miR-20b-5p, miR-17-5p, miR-130a-3p, miR-18a-5p, miR-195-5p, miR-486-5p, and miR-93-5p in the serum of BC patients with recurrence when compared to those without recurrence [154].

Furthermore, exosome miRNA profiling in plasma, specifically in patients with ductal carcinoma in situ (DCIS) and primary BC patients with recurrence compared to healthy counterparts, revealed distinctive expression patterns. Higher levels of miR-16 and miR30b were associated with recurrent patients, while miR-93 exhibited elevated levels in DCIS patients [155]. This underscores the potential of exosome miRNAs as discerning markers for distinguishing recurrent BC from the early stages of the disease.

In a separate investigation profiling 35 differentially expressed miRNAs in plasma exosomes from early-stage BC patients, miR-375 and miR-24–2-5p were identified as highly abundant miRNAs negatively correlated with patient survival. Conversely, significantly down-regulated miR-548b-5p, miR-655-3P, and miR-376b-5p were found to be positively correlated with survival outcomes [156]. Bao et al. employed a combined approach, integrating genomic instability (GI) analysis with exosome miRNA profiling, leading to the identification of three exosome miRNA signatures (miR-421, miR-128–1, and miR-128–2) in the serum of BC patients, associated with poor prognosis [157].

Baldasici et al. propose a non-invasive method for early BC diagnosis, isolating miRNAs in tumor-derived exosomes (TEx). They identify exosomal miRNAs associated with BC metastasis, offering a potential array of biomarkers for various metastatic scenarios [158]. Risha et al., utilizing nano LC–MS/MS, profiled 726 uniquely expressed proteins in TNBC cells, identifying Glypican-1 (GPC-1), glucose GLUT-1, and a Disintegrin and metalloproteinase domain-containing protein 10 (ADAM10) as potential biomarkers located on the membrane surface of exosomes and up-regulated compared to a non-tumorigenic epithelial breast cell line [159]. A multiplexed cantilever array showed that GPC-1 in exosomes released from BC cell lines demonstrated high sensitivity and throughput in real-time acquisition [160].

In the realm of inflammatory BC, a rare and aggressive malignancy often misdiagnosed as mastitis, EVs extracted from patient plasma have unveiled the presence of three specific miRNAs (miR-181b-5p, miR-222-3p, and let-7a-5p). The diagnostic potential of these miRNAs, indicated by a high area under the curve (AUC > 0.9) in receiver operating characteristic curve analysis, positions them as promising diagnostic biomarkers for inflammatory BC. This is particularly crucial for accurate and timely diagnosis, given the ease of misdiagnosis as mastitis, which can result in delayed treatment [161, 162]. Exploring the realm of metabolites, Buentzel et al. identify eight metabolites, including lysoPCaC26:0 and PCaaC38:5, strongly associated with poor prognosis in BC patients [163]. Furthermore, studies by Cai et al. and Chen et al. delve into the potential of mRNAs and phosphorylated proteins in EVs as biomarkers for BC, offering diagnostic and screening possibilities [139, 164].

Recent advancements in exosome screening methods, including microfluidic chips, surface-enhanced Raman scattering nanotags, and DNA aptamer-mediated microfluidics, offer simplified and time-saving approaches to profile exosome Epithelial Cell adhesion Molecule (EpCAM) and HER2 proteins, particularly for the diagnosis of HER2 + BC. While these findings collectively highlight the diagnostic value of exosome protein biomarkers in BC, further validation through large-scale studies involving independent clinical samples is imperative to confirm their clinical significance [147, 148, 160, 165].

These findings collectively emphasize the utility of exosome-derived miRNAs as a valuable tool for BC prognosis, providing insights that are pivotal for refining clinical strategies and enhancing patient outcomes.

## EV-mediated drug delivery in BC

### Harnessing EVs for drug delivery in BC

#### Choice of parent cell for EV production

As potential carriers for targeted drug delivery in nanotechnology, EVs offer advantages such as lower molecular weight, good bioavailability, lower toxicity, and tissue-specific receptor coating. Sources of EVs for drug delivery include red blood cells, macrophages, DCs, and platelets, with studies demonstrating their effectiveness against specific cancer cells including BC cells [166].

#### Extraction and characterization of EVs

To extract EVs, various separation methods are employed, focusing on preserving their integrity and characteristics. Ultracentrifugation, a common method, may cause mechanical damage and affect purity [167]. Ultrafiltration, based on size, is rapid but prone to clogging. Tangential flow filtration, an automated alternative, can result in membrane interactions. Studies, such as one by Busatto et al., compare methods, indicating that Tangential flow filtration is superior in yield and impurity removal for BC cell cultures [168].

Blind elute chromatography, based on size/affinity, has limited sample capacity. Precipitation methods offer convenience but may introduce impurities. It is crucial to choose a separation method that yields high-purity EVs at minimal cost and ensures safety, considering the diverse pros and cons of each technique in this evolving field.

**Fig. 3:**
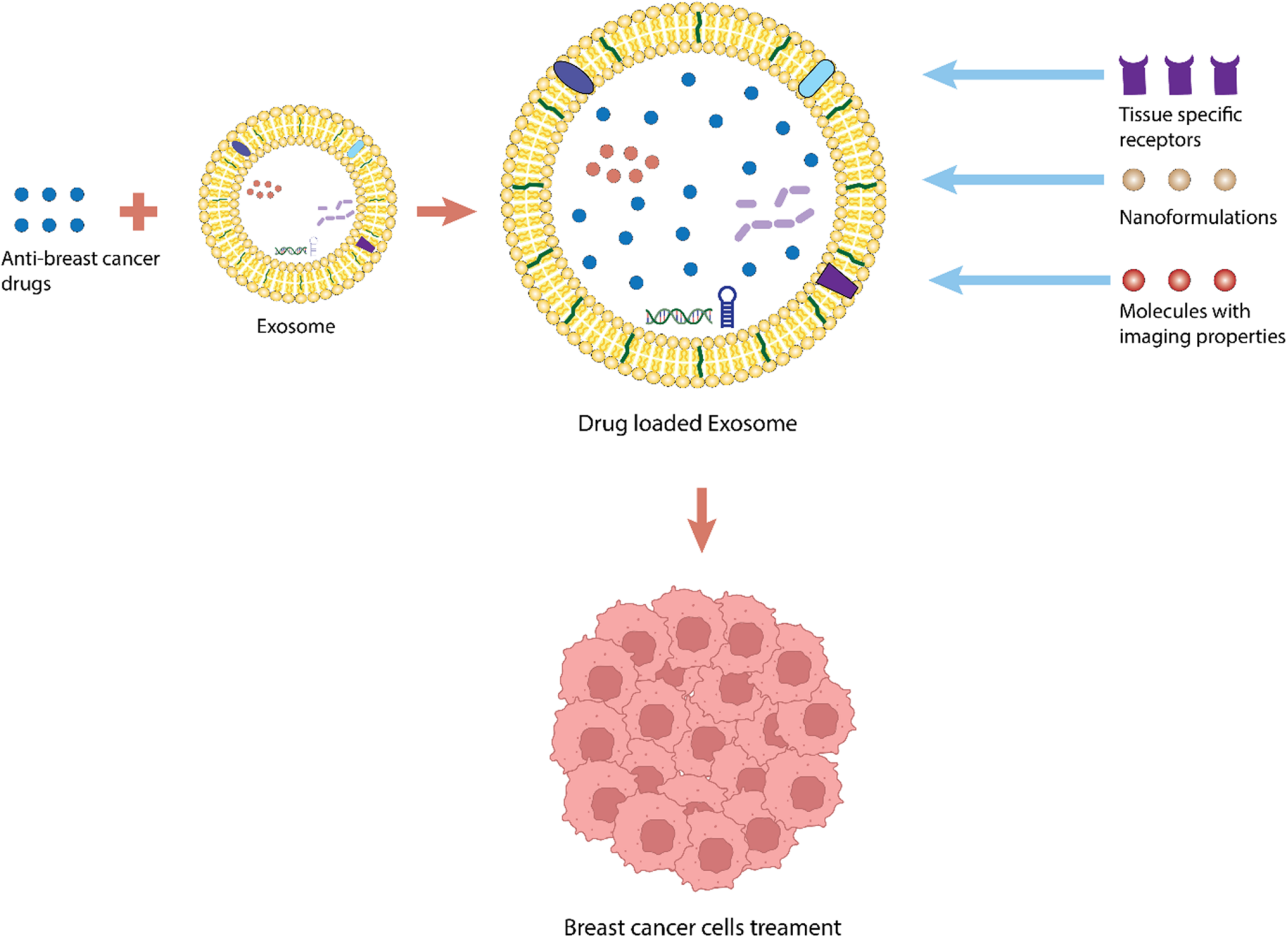
Illustration of Delivery of Anticancer Drugs for Treatment of Breast Cancer. This figure depicts the delivery of anticancer drugs for breast cancer treatment. Exosomes, nanovesicles secreted by cells, efficiently transport drugs actively or passively loaded within their cargo. Labeling of extracellular vesicles enables their tracking in systemic circulation and targeted delivery to specific tissues

As carriers for drug delivery, EVs show potential in transporting therapeutic substances such as nucleic acids, and anti-breast cancer drugs (Table 2**.** [169–179] [180, 181]) [182, 183]. Furthermore, they can be labeled with tissue-specific receptors and molecules or devices with imaging properties such as biosensors (Fig. 3) [184, 185]. This enables their tracking in systemic circulation and targeted delivery of drugs to specific tissues. The suitability of EVs as delivery agents stems from their minimal immunogenicity and favorable biocompatibility. During the process of cellular growth, intercellular communication persists through EVs. Within this stage, cells have the capacity to internalize drugs and subsequently secrete them through these vesicles. Consequently, the vesicles serve as carriers for loading the drugs.

**Table 2:** Variability in Drug Loading within EVs for Breast Cancer Therapy: Comprehensive Analysis of Cargo Types, isolation method, loading method and EV Sources

Cargo	Source of EV	Isolation method	Loading method	Outcome of the study	References
Doxorubicin	Immature dendritic cells (mouse)	Ultracentrifugation	Electroporation	Purified exosomes from imDC loaded with Dox had an encapsulation efficiency of 20%. Intravenous Injection of loaded Dox resulted in tumor growth inhibition without significant toxicity	[169]
Doxorubicin	MCF-7 breast carcinoma cells (human) ADR/MCF-7 doxorubicin resistant breast carcinoma cells (human)	–	–	–	[170]
VEGF siRNA	Primary dendritic cells (mouse)	Ultrafiltration	Electroporation	AS1411-EVs loaded with miRNA let-7 selectively targeted tumor tissues in tumor-bearing mice and inhibited tumor growth. Also, the modified EVs were well tolerated and showed no evidence of nonspecific side effects or immune response	[171]
Doxorubicin	HEK293 cell	Total Exosome Isolation Kit (Invitrogen)	Transfection	293T cell-derived exosomes are safe and suitable for use as in vivo drug delivery vehicles	[172]
Paclitaxel	RAW 264.7 macrophages	Double emulsification	Incubation	The study showed the high efficiency of a macrophage-mediated delivery system and showed its advantage over traditional drug delivery methods	[173]
Doxorubicin	Grape fruit	Centrifugation	Sonication	–	[174]
Doxorubicin	RBC	Ultracentrifugation	Fusion of EVs with functionalized liposomes triggered by polyethylene glycol (PEG) to form hybrid vectors	Hybrid EVs Improved cellular delivery of chemotherapeutic compounds, enables the biocamouflage of liposomes and drug delivery	[175]
miRNA 100, miR-379	Mesenchymal cell		Exosomal transfer of miR-100 from Human mesenchymal stem cells (MSC)	A novel method underlying the paracrine effects of MSC-derived exosomes and modulation of vascular responses in breast cancer cell environments	[176]
miR-142-3p inhibitor	Mesenchymal cell	Review article	–	–	Lee and Dutta [177]
Lamp2b fusion proteins with peptides (176)	FBS-derived exosomes	Ultracentrifugation	Incubation	Glycosylation motif GNSTM-tagged peptides enhance targeted delivery of exosomes to neuroblastoma cells	[178]
CpG DNA-modified exosomes (CpG-SAV-exo	Tumor cell-derived exosomes	Ultracentrifugation	Incubation	Enhanced tumor antigen presenting capacity, useful for cancer immunotherapy	[179]
Paclitaxel	Mesenchymal stroma/stem-like cells (MSC) derived exosomes	Centrifugation	Incubation	Effective in inhibiting primary tumor growth and reducing distant organ metastases, Effectively targeting primary tumors and metastases by reducing side effects	[180]
Paclitaxel and 5-Fluorouracil	Folic acid (FA) functionalized bovine milk derived exosome	Ultracentrifugation	Sonication after dissolving in methanol/pbs (phosphate buffered saline)	Enhanced efficacy against breast cancer. Significant decreases in IC50 observed. Higher apoptotic index and better control over cell migration	[181]

#### Loading of drugs into EVs

There are two main approaches to loading drugs into EVs: pre-loading, which involves loading drugs before EV isolation, and post-loading, where drugs are loaded after isolation. The pre-loading approach allows for the continuous and straightforward generation of EVs loaded with cargo without compromising the integrity of the vesicle membrane. The common methods for pre-loading are co-incubation, transfection, and ultrasound-stimulated microbubbles (USMB).

In the process of co-incubation, drugs are co-incubated with parent cells under specific conditions, facilitating their loading into cells through spontaneous interaction with the lipid bilayer. The drugs are then secreted along with the EVs. Despite its simplicity, the method often exhibits low loading efficiency, influenced by factors such as drug characteristics, concentration gradient, and the type of parent cells [186]. Various chemotherapeutic drugs, particularly hydrophobic ones like Doxorubicin (DOX) and Paclitaxel (PTX), have been successfully loaded by co-incubation [187–191]. EVs loaded with PTX have demonstrated encouraging outcomes in effectively targeting and treating BC [166].

Recent investigations have indicated that inducing apoptosis in healthy cells through Ultraviolet (UV) exposure enhances the efficiency of loading co-incubated drugs or nanoparticles into EVs [170, 192, 193]. Notably, Fukuta et al. discovered that treating cells with low electrical currents could activate intracellular signaling, promote endocytosis of macromolecules, and enhance the secretion of EVs [194]. By means of cellular transfection, parent cells can be induced to overexpress therapeutic cargo, encompassing RNAs and proteins, subsequently packaging them into EVs. ExoIL-12, incorporating Prostaglandin F2 receptor negative regulator (PTGFRN), has made history as the pioneering engineered exosome candidate drug to progress into clinical trials, marking a significant milestone in the realm of medical research [195]. The use of lipid-based transfection presents advantages in terms of heightened repeatability and simplicity. Nonetheless, this approach is not without its inherent challenges, notably, the presence of low transfection efficiency and a significant dependence on the vitality of the cells [196, 197].

USMB stimulation has been demonstrated to induce the release of EVs from cancer cells. Yuana et al. employed this method to generate drug-loaded EVs [198]. They loaded the fluorescent dyes cell tracker green (CTG) and bovine serum albumin coupled with fluorescein isothiocyanate (BSA FITC) into human umbilical vein endothelial cells (HUVECs) using varying ultrasound acoustic pressures. Notably, at 0.6 MPa, the BSA-FITC signal intensity in HUVECs reached its peak, surpassing both untreated conditions and treatments at 0.7 and 0.8 MPa. The BSA-FITC signal intensity induced by USMB exceeded that of the untreated group. It is crucial to note that USMB may not be suitable for substances prone to quenching and entrapment in the endosomal-lysosomal degradative pathway post-uptake. Despite its effectiveness in producing cargo-loaded EVs, a notable drawback of USMB lies in its susceptibility to entrapment within cellular organelles.

In the realm of drug loading into isolated EVs, the post-loading approach emerges as a highly customizable strategy, offering advantages over pre-loading by minimizing the incorporation of interfering substances [199]. Post-loading encompasses two main categories: passive loading and active loading. Passive loading involves the co-incubation of EVs with high drug concentrations, allowing drugs to interact with the lipid bilayer and diffuse passively into the vesicle's lumen [200]. While this method is simple and retains membrane integrity, limitations include low loading efficiency and restricted applicability to hydrophobic compounds [201]. For instance, Saari et al. demonstrated a mere 9.2% loading efficiency of the hydrophobic drug PTX into EVs through co-incubation [201]. The duration of co-incubation varies, with curcumin achieving loading in as little as 5 min, while DOX typically requires overnight incubation [202].

Active loading becomes necessary when cargo cannot passively diffuse through EV membranes. This approach involves physical induction and chemical induction methods [203]. Physical induction commonly entails the immediate disruption of EV membranes through external forces, while chemical induction relies on transfection agents to aid cargo loading without causing harm to the integrity of EV membranes [204, 205]. Physical induction methods, including electroporation, sonication, extrusion, and freeze–thaw cycles, temporarily affect membrane permeability to facilitate cargo entry [206]. Electroporation, using high-intensity pulsed electric fields, induces transient permeable pores on EV membranes, enhancing cargo entry, particularly effective for siRNA loading [207, 208]. However, concerns arise regarding the impact on Zeta potential and colloid stability, which may lead to siRNA and EV aggregation [209]. To mitigate this, a trehalose pulse medium has been developed to enhance colloidal stability and reduce aggregation caused by electroporation [209].

Sonication increases membrane permeability by deforming EV membranes, promoting cargo diffusion, with higher loading efficiency compared to co-incubation and electroporation [166, 206, 210]. Haney et al. utilized EVs sourced from macrophages in a targeted approach against TNBC through the application of the sonication method [166]. These EVs were employed as carriers for the drug delivery of PTX and DOX subsequent to incubation (DOX) or sonication (PTX). The resultant EVs loaded with DOX and PTX exhibited a spherical morphology with a uniform size distribution. Remarkably, these EVs demonstrated noteworthy attributes, including effective intracellular accumulation, successful drug accumulation within cancer cells, minimal immunogenicity, and heightened stability. Despite its efficacy, sonication may compromise EV structure due to mechanical shear force, prompting recommendations for execution in an ice bath [211].

Extrusion involves passing the EV-cargo mixture through nanoscale aperture membrane filters, disrupting EV membranes, and aiding cargo loading [174]. While extrusion produces homogeneous EVs, it may alter Zeta potential and membrane proteins [206].

Freeze–thaw cycles alternate rapid crystallization and thawing, temporarily disrupting EV membranes and facilitating cargo entry [212–214]. However, this approach tends to increase EV size, inducing aggregation [214].

Chemical induction methods utilize saponins or transfection agents to enhance cargo loading without damaging EV membranes [215, 216]. Although saponin significantly enhances loading efficiency, its potential for hemolysis necessitates strict concentration control and additional EV purification [216], Transfection reagents such as liposomes and EXO-Fect expedite cargo loading into isolated EVs [208, 217]. Nevertheless, residual transfection agents may pose toxicity concerns, limiting their application [218]. An alternative method employs calcium chloride for miRNA transfection into EVs, demonstrating similar efficacy to electroporation while ensuring simplicity and stability [219]. Thus, the post-loading approach offers a versatile means for drug loading into EVs, with passive and active loading methods presenting distinct advantages and considerations. The choice of method should align with the specific cargo characteristics and desired outcomes in drug delivery applications [199].

#### Advantages and disadvantages of EVs as delivery systems

EVs represent a promising drug delivery system owing to their resemblance to parent cells, facilitating intercellular communication and disease microenvironment modulation [220]. Unlike synthetic nanoparticles, EVs possess the remarkable ability to traverse both extracellular and intracellular barriers, enabling efficient transport of beneficial biomolecules across distant cells [221, 222]. Notably, EVs exhibit low toxicity, minimal immunogenicity, and exceptional stability, delivery efficiency, and biocompatibility, enhancing their therapeutic potential [223]. Their unique capacity to traverse the blood–brain barrier further underscores their applicability in targeting specific sites within the brain [224]. Leveraging endogenous cellular mechanisms, EVs can safeguard and deliver functional cargo, rendering them highly appealing as therapeutic agents. Nevertheless, challenges persist in establishing a consistent biochemical strategy for their clinical therapeutic utilization. Moreover, hurdles exist in achieving effective brain-targeted drug delivery via EVs. Addressing these challenges necessitates further research to elucidate the underlying mechanisms governing the therapeutic efficacy of EVs.

### Application of EV-mediated drug delivery in treatment of BC

Challenges in treating BC, including drug tolerance, high toxicity, and other mechanisms leading to treatment failure, has prompted the exploration of novel therapies and drug delivery technologies [3]. Precision oncology, with its potential to identify molecular biomarkers, has garnered attention in this context. Researchers are increasingly interested in exosomes (which are endosome-released nanometer-sized EVs) as a promising avenue for targeted drug delivery and a novel cancer vaccine [225]. exosomes offer advantages over other nanoparticle drug delivery systems, such as liposomes or polymeric nanoparticles, due to their biocompatibility, biodegradability, and lower toxicity [226]. Additionally, exosomes exhibit limited immunogenicity and cytotoxicity, and their ability to traverse anatomical barriers enhances their potential as drug carriers [227, 228].

Clinical trials are underway to explore the therapeutic potential of exosomes in BC, with various exosome-based therapies showing promise in improving chemotherapy effectiveness. exosomes have been employed to deliver chemotherapeutic drugs such as PTX and doxycycline (DOX) [229, 230]. The loading of DOX in exosomes not only reduces cardiotoxicity but also enhances its efficacy compared to traditional administration [203, 231]. Similarly, PTX-loaded exosomes exhibit greater efficiency in inhibiting cancer cell growth than free PTX and liposomal PTX [232]. Hybrid exosomes (HE) formed by fusing exosomes with liposomes show great results in improving PTX loading capacity for TNBC chemotherapy [233].

Exosomes are believed to be involved in multiple stages during invasive processes, likely contributing to early steps in metastasis [234]. Furthermore, exosome-mediated delivery of tumor-secreted miR-105 selectively destroys tight junctions and the integrity of natural barriers, enhancing metastasis in BC [114]. Collaborative research has shown that the ASPH network regulates designated exosomes to enable the delivery of a pro-oncogenic secretome, facilitating long-distance metastasis. At the same time, numerous studies have revealed the influential role of exosomes in reducing metastasis activity in BC.

In BC xenografts, sulfisoxazole, an oral antibiotic with anti-tumor and antimetastatic properties, interferes with endothelin receptor A and decreases exosome release, ultimately inhibiting progression and metastasis in BC cells [235]. Additionally, the tumor suppressor nischarin has been found to regulate early metastatic events in BC, with further research demonstrating its novel role in preventing BC cell motility and tumor growth by regulating Rab14 activity and secreting exosomes that control tumor malignancy [236, 237]. Another study suggested that antisense non-coding mitochondrial RNA could be a novel target for BC therapy, with exosomes derived from knockdown cells reducing tumorigenic properties and inhibiting the development of BC metastatic niches [238].

Exosomes-mediated siRNA has shown promise as a therapeutic strategy to inhibit metastasis in postoperative BC patients [239]. Exosomes serve as carriers for nucleic acid molecules and can be genetically engineered to deliver specific DNA or RNA molecules. SEV-mediated delivery of miRNAs, such as miR-142-3p and let7c-5p, has demonstrated significant suppression of BC cell proliferation and migration [240, 241]. Engineered exosomes loaded with miR-let-7a efficiently target Epidermal Growth Factor Receptor (EGFR) -expressing cells, resulting in tumor growth inhibition [242]. Moreover, an EV-based drug delivery system demonstrated a synergistic anti-tumor effect, enhancing tumor-killing efficiency by 15% through the promotion of the drug's tumor-targeting capabilities [243]. The microbiota has been recognized for its crucial role in cancer progression and treatment [244, 245]. An et al. conducted a study highlighting the considerable enhancement of the therapeutic effect of tamoxifen on ER + MCF7 cells through the combination of Klebsiella pneumoniae-derived EVs and tamoxifen. This combination downregulated cyclin E2 and p-ERK expression, providing an effective approach [246].

Microbiome-mediated regulation of estrogen metabolism, referred to as the estrogenome, has been implicated in BC development. An et al. compared the EVs profiles of blood microorganisms from BC patients and healthy controls, revealing that Staphylococcus aureus -derived EVs influenced the efficacy of tamoxifen by modulating extracellular signal-regulated kinase (ERK) and Protein kinase B -related signaling pathways. The synergistic combination of S. aureus-derived EVs and tamoxifen impeded the growth of ER + BC cells [247]. Furthermore, exosomes facilitate combinational therapy, as seen in the co-delivery of miR-159 and DOX for TNBC therapy [225]. exosomes also deliver long non-coding RNAs (lncRNAs), such as DARS-AS1, suppressing TNBC cell growth and liver metastasis [248]. Utilizing a tissue engineering approach, Gong et al. developed EVs containing therapeutic doses of adriamycin and cholesterol-modified miR-159, resulting in effective anti-tumor effects against TNBC cells [225].

The pharmacologically important characteristics of exosomes have led to the development of nanoparticles based on cell-derived exosomes through tissue engineering, significantly contributing to tumor treatment [249–251]. Shi et al. created a synthetic multivalent antibody retargeted exosome platform using tissue engineering, modifying exosomes to target human-derived Clusters of Differentiation (CD3) and HER2 proteins [183]. This approach efficiently targeted HER2-expressing BC cells by recruiting CD3 + -expressing cytotoxic T cells, demonstrating a promising strategy against HER2 + BC.

A novel therapeutic strategy involving antibodies targeting exosomes has demonstrated efficacy both in vivo and in vitro, as macrophages internalized and eliminated antibody-tagged cancer-derived exosomes, resulting in decreased metastatic incidence [252]. Interestingly, specific cancer cell-released exosomes have been found to inhibit lung cancer cell proliferation and migration in a recent study, suggesting their potential role in treating other cancer cells [253]. These exosomes, released from tumor cells, were exploited as miRNA-126 protective nanocarriers, inhibiting the Protein Kinase B signaling pathway and suppressing pulmonary tumor cell metastatic ability [253]. In addition, Chang et al. demonstrated that endocytosis of EVs secreted from Wharton’s Jelly MSCs (WJ-MSCs) into TNBC cells significantly reduced proliferation potential, stem cell characteristics, tumor formation capacity, and metastatic capacity under hypoxic conditions [254]. This suggested the potential therapeutic effects of targeting TNBC with MSC-derived EVs. Further mechanistic analysis revealed that WJ-MSC-secreted EVs attenuated the tumorigenic ability of TNBC cells and prevented immunosuppression in the TME by transferring miR-125b and inhibiting HIF1a signaling pathway-related protein expression [254].

Targeting the polarization of TAMs from the M1 to M2 phenotype has proven to be an efficacious strategy in BC treatment [255–257]. It is noteworthy that the M1 phenotype of TAMs exhibits pro-cancer effects, while the M2 phenotype exerts cancer-suppressive effects. In a novel approach, Zhao et al. proposed a system to load docetaxel into M1 TAM-derived exosomes, leading to the conversion of the M1 phenotype to the M2 phenotype [258]. This innovative strategy significantly improved the anti-cancer effect with minimal side effects.

Exosomes are explored not only for drug delivery but also as potential components of tumor vaccines [259]. Topotecan-induced exosomes containing DNA have been investigated for their ability to activate DCs and stimulate immune responses against BC cells [260]. exosomes from DCs, when engineered with antigens and adjuvants, show promise in initiating precise immune responses against tumor cells, presenting a fundamental approach for developing DC vaccines [261].

Exosomes exhibit heterogeneity based on cell conditions, providing various possibilities. Engineering exosomes with ligands specific to targeted cancer cells is crucial for their effective use in cancer treatment. Bioengineered exosomes expressing designed ankyrin repeat proteins (DARPins) on their membrane surface demonstrate specific binding to HER2, showcasing the potential for targeted drug delivery [207]. Engineered exosomes with CD3 and EGFR expressions induce T cell cross-linking and elicit antitumor immunity both in vitro and in vivo [262]. Furthermore, exosomes engineered with anti-human CD3 and anti-human HER2 antibodies redirect and activate cytotoxic T cells toward attacking HER2-expressing BC cells [183].

The therapeutic potential of exosomes in cancer, particularly BC, is promising, and ongoing clinical trials are investigating their efficacy [263]. However, challenges remain, and further breakthroughs are needed to optimize these novel therapeutic approaches in vivo. A comprehensive understanding of exosome biology is imperative to expedite vectorization in BC patient treatment.

## State of clinical trials of EVs in BC theranostics

The potential of EVs has been well described in many diseases, including BC. Due to their dynamic characteristics, exosomes are currently employed as biomarkers and drug-delivery vehicles in clinical trials. Exosomes used in clinical trials are derived from two primary sources- human cells/samples and plants. The most common human source of clinical trial exosomes is MSCs [264]. We will explore the different applications of exosomes in clinical trials below.

### Exosome as a biomarker in clinical trials

The biomarker application of exosomes is the most explored potential in clinical trials. This is based on the knowledge that exosomes carry a complex selection of molecules from one cell to the other. Hence, observing exosome components at a given time of release can help characterize the state of the cell of origin. Transcriptomics and proteomics changes in the cargo of exosomes are analyzed and observed [265]. Thus, exosomes provide an avenue for measuring cell status and function, making it valuable tool for diagnostics [266]. Differential expression of miRNA on exosomes has been consistently observed in some carcinomas, giving a potential for using exosomal miRNA as a biomarker. A clinical study conducted on patients with stage 1 Non-small cell lung cancer (NSCLC) used miRNA profiles of exosomes to differentiate between adenocarcinoma (AC) and squamous cell carcinoma (SCC). AC-specific and SCC-specific exosomal miRNA were characterized by next-generation sequencing (NGS), showing that exosomal miRNA constitutes a sensitive biomarker to classify early-stage cancers [267]. In another study, three miRNAs (miR-181b-5p, miR-222-3p, and let-7a-5p) were selectively identified to be upregulated in small EVs from women with inflammatory BC [162]. Similarly, Wang et al. identified a correlation between the downregulation of the exosomal miRNA, miR-363-5p, and lymph node metastasis in BC patients [149]. Other clinical studies have also detected specific changes in exosomal miRNA in BC patients. There is a need for further research on exosomal miRNA before their use as a biomarker for BC [23, 158]. Many clinical trials employ EVs as a predictive or prognostic marker in BC (Table 3**.** [268]). A clinical trial (currently withdrawn) has explored the potential of TEx as a predictive and prognostic biomarker in patients receiving Neoadjuvant Chemotherapy (NCT01344109). Another clinical trial evaluates the potential of TEVs and tumor-derived circulating tumor DNA as predictive markers for drug response and metastasis in early BC patients (NCT05955521). Molecular changes in exosomes isolated from tumors and liquid biopsies (Urine, Blood, Tears, etc.) have also been used in the trial to track resistance development to Palbociclib in metastatic BC patients(NCT04653740). In an ongoing trial, EVs from blood are used to characterize the genomic signature of metastatic BC patients (NCT04258735). The genomic changes in exosomes in this trial are correlated with patient survival. Another trial employed microvesicles isolated from BC patients' Cerebrospinal fluid (CSF) to predict leptomeningeal metastasis (NCT03974204). In an ongoing clinical trial, HER 2 expression in the TEx and blood-derived exosomes are compared to highlight the potential of liquid biopsy measurement in HER 2 positive BC patients (NCT04288141). The immunomodulatory potential of pembrolizumab is also being assessed using serum-derived EVs in TNBC patients (NCT02977468). Other ongoing or completed clinical trials assess EVs' biomarker potential in BC [1].

**Table 3:** Summary of Clinical Trials on the Use of EVs as biomarkers for Breast Cancer

Trial number	Exosome source	Trial objective	Outcome/expected outcome	Status	Link
NCT01344109	Tumor	To use exosomes as a diagnostic and prognostic marker for breast cancer patients receiving Neoadjuvant Chemotherapy	–	Withdrawn	https://clinicaltrials.gov/study/NCT01344109?term=exosomes&cond=Breast%20Cancer&city=&rank=1
NCT05955521	Tumor	To evaluate circulating tumor DNA (ctDNA) and tumor-derived exosomes as a predictive and prognostic marker in early BC patients	ctDNA or exosomes could be used as a marker for predicting recurrence in early BC patients receiving Neoadjuvant Chemotherapy	Active Not Recruiting	https://clinicaltrials.gov/study/NCT05955521?term=exosomes&cond=Breast%20Cancer&city=&rank=2
NCT04653740	Tumor & Liquid Biopsy	To assess longitudinal changes in exosomes to assess resistance to Palbociclib in metastatic BC patients	Changes in the molecular signature of exosomes after Palbociclib will explain resistance development	Unknown	https://clinicaltrials.gov/study/NCT04653740?term=exosomes&cond=Breast%20Cancer&city=&rank=5
NCT04258735	Blood	Genomic analysis of metastatic breast cancer patients	Genetic changes will be associated with patient survival	Ongoing	https://clinicaltrials.gov/study/NCT04258735?term=exosomes&cond=Breast%20Cancer&city=&rank=6
NCT03974204	Cerebrospinal Fluid (CSF)	Using microvesicles from the CSF of breast cancer patients to diagnose leptomeningeal metastasis	Comparison between the proteomic profile of the isolated microvesicles can signal leptomeningeal metastasis	Withdrawn	https://clinicaltrials.gov/study/NCT03974204?term=exosomes&cond=Breast%20Cancer&city=&rank=7
NCT04288141	Tumor & Blood	Comparing HER2-HER3 dimer expression in tumor and blood-derived exosomes in HER 2 positive BC patients receiving anti-HER 2 therapy	HER2 expression in the blood-derived exosomes will be similar to the expression level in tumor-derived exosomes to avoid invasive diagnosis of HER 2 BC	Ongoing	https://clinicaltrials.gov/study/NCT04288141?term=exosomes&cond=Breast%20Cancer&city=&page=2&rank=11
NCT02977468	Serum	To assess response to pembrolizumab in TNBC patients	The administration of pembrolizumab in patients will alter the expression of immune-tolerant markers	Ongoing	https://clinicaltrials.gov/study/NCT02977468?term=exosomes&cond=Breast%20Cancer&city=&page=1&rank=8

Information on this table was gotten from the following link: https://clinicaltrials.gov/search?term=exosomes&cond=Breast%20Cancer&city = on the 7th of December, 2024 at 17:37

### Exosome as a drug delivery tool in clinical trials

Unlike biomarker applications, the use of EVs for drug delivery for BC is still limited. This limitation is mainly due to concerns relating to the characterization and safety of the EVs. However, few studies have employed EVs for drug delivery [269]. A phase 1 study completed in 2022 employed plant-derived exosomes to deliver curcumin to healthy subjects and patients with colon cancer (NCT01294072). Similarly, another clinical trial is being conducted focused on using EVs to deliver curcumin for Inflammatory Bowel Disease (IBD) (NCT04879810). The safety of genetically engineered EVs expressing CD24 for moderate-to-severe Coronavirus Disease 19 (COVID-19) is being tested (NCT04747574). An ongoing Phase 1 trial tests the tolerance and efficacy of MSC-derived EVs loaded with KrasG12D siRNA in pancreatic cancer patients (NCT03608631). In addition, miR-124 loaded exosomes derived from MSCs have been administered in Phase 1 to ameliorate acute brain injury (NCT03384433).

## Challenges of EV translation in BC therapy

Although EVs have a tremendous physiological advantage as a nano-sized carrier for drugs in BC, their use in therapy is currently limited. This is due to some technical problems with the standardization, isolation, and purification of EVs. These limitations include:

### EV heterogeneity

For EVs to be applicable in loading medications for clinical use, there must be a detailed understanding of the biogenesis and composition of EVs. However, due to their heterogeneous nature, there has been a lack of complete knowledge of EV characteristics. The fact that EVs derived from the same cell can have different molecular compositions has made it complex to standardize EVs for therapeutic use [270]. In addition to the intrinsic diversity of EVs, the isolation method used can impact heterogeneity in EVs by altering their physicochemical features and purity states. Hence, before EVs can be used as drug carriers, it is essential to understand their heterogeneity and the factors contributing to their diversity [271].

### Choice of the parent cell

Although many cells have been shown to secrete EVs, there is still the question of what the ideal cell to extract EVs for BC therapy is. The lack of credible answers to this question has played a part in the factors limiting EV use in clinical trials. This is because the intrinsic behavior of isolated EVs depends mainly on the parent cells—for example, the cytotoxicity effect observed in EVs isolated from immune cells [272]. The property of the cell also determines the efficiency of loading drugs to the EVs in indirect loading techniques. Therefore, further characterization of the best cell of origin is needed for isolating cells for BC therapy [271, 273].

### Loading procedure employed

As previously discussed, the loading techniques determine the efficiency of drugs loaded on the EVs. Apart from the loading efficiency, the methods employed can also affect EVs' integrity, resulting in biological limitations for EVs. Loading techniques can induce modifications affecting EVs' quality, purity, and storage conditions. Therefore, the most efficient loading technique for EVs must be established for loading drugs for BC therapy [271, 274].

Genetically engineering parental cells for cargo loading of EVs ensures RNA molecules are properly encapsulated and protected within the EV lumen, unlike post-loading methods which may lead to RNA localization on the EV surface [275]. However, monitoring cargo-loading efficiency in pre-loaded EVs is challenging, and potential changes in EV composition due to recombinant protein overexpression cannot be overlooked. Similarly, damage to EVs during post-loading methods like electroporation or sonication is a concern. Hence, the choice between pre-loading and post-loading methods for miRNA loading depends on research goals. Electroporation is currently the most popular method for miRNA loading due to its simplicity and speed [276, 277].

### Route of administration

EV-mediated drug delivery aims to deliver drugs to the target cell or tumor site. For targeted delivery, a good understanding of the best route of administration of EVs is essential. This is because various routes can induce EV clearance differently. For appropriate translation of EVs for BC therapy, a complete understanding of the best route to administer designed EVs [271]**.**

### Storage of EVs

Preserving the therapeutic properties of extracted EVs poses a challenge due to their sensitivity to storage conditions. EVs undergo changes in surface characteristics, morphology, and protein content during storage [278]. Temperature fluctuations affect EV stability, with lower temperatures (-70°C to -80°C) proving optimal for preservation [279–281]. Aggregation, particularly at -70°C, threatens EV structure and function [282]. Maintaining an acidic pH environment enhances EV uptake [283], while freeze–thaw cycles impact EV concentration but not uptake significantly [281, 284]. Balancing these factors presents a challenge in ensuring effective EV uptake while minimizing concentration reduction.

## Limitations

While this review aimed to provide a contemporary perspective on EV-mediated drug delivery in BC theranostics, several limitations should be noted. Firstly, the potential for publication bias exists, as only published articles were included. Additionally, the review focused on English-language publications, potentially excluding relevant studies in other languages.

The choice to use PubMed, SCOPUS, and Google Scholar, while providing a comprehensive overview, may have overlooked relevant studies in other databases. Despite efforts to ensure completeness, some studies may have been inadvertently missed during the literature search.

The heterogeneity in study designs, methodologies, and outcome measures among included studies may pose challenges in synthesizing data. Furthermore, the rapid evolution of EV research may render certain aspects of this review subject to updates as the field progresses. Acknowledging these limitations is crucial for interpreting the findings and guiding future research directions in this dynamic field.

## Conclusion

As BC stands as the most frequently diagnosed cancer globally, there is a need for proper theranostics. The article comprehensively analyses EVs and highlights their crucial role in BC diagnosis and drug delivery. The review has shown that EVs play a vital role in shaping the TME and influencing essential aspects of cancer. However, research has shown that EVs have great potential in BC theranostics, specifically as biomarkers and drug delivery vehicles. Studies showed that this discovery is a significant step in advancing personalized medicine. Nevertheless, the challenge of early diagnosis persists, and EVs offer significant potential as non-invasive biomarkers that could enhance the sensitivity of existing diagnostic techniques. It is worth mentioning that the presence of circulating exosomal miRNAs, long noncoding RNAs, and proteins in EVs holds potential for early detection, disease monitoring, and evaluating treatment effectiveness. In addition, the biocompatibility of EVs enables them to function as natural carriers for drug delivery, effectively overcoming the limitations associated with traditional treatment methods.

Based on the findings of the review, it is evident that EV-mediated drug delivery has the potential to significantly enhance drug penetrance, stability, and cellular uptake in specific areas. This, in turn, can significantly improve therapeutic effectiveness while minimizing any unintended toxic effects. Continued exploration and progress in this field could lead to the creation of advanced diagnostic tools and specialized treatments, ultimately enhancing the management of BC with greater effectiveness and personalization.

## Data Availability

No datasets were generated or analysed during the current study.

## Abbreviations

AC: Adenocarcinoma
ADAM10: A Disintegrin and metalloproteinase domain-containing protein 10
ALN: Axillary lymph node
ASC: Adipose-derived stem cell
AUC: Area under the curve
AURKB: Aurora kinase B
BSA FITC: Bovine Serum Albumin coupled with fluorescein isothiocyanate
CA: Cancer antigen
CAA: Cancer associated adipocyte
CAF: Cancer-associated fibroblast
CD: Clusters of differentiation
CEA: Carcinoembryonic antigen
COVID-19: Coronavirus disease 19
CSF: Cerebrospinal fluid
Cx46: Connexin-46
CTG: Cell tracker green
DC: Dendritic cell
DCIS: Ductal carcinoma in situ
DARPins: Designed ankyrin repeat proteins
DOX: Doxorubicin
ECM: Extracellular matrix
EDIL3: Epidermal growth factor-like repeats and discoidin i-like domains 3
EGFR: Epidermal growth factor receptor
EpCAM: Epithelial cell adhesion molecule
ER: Estrogen receptor
ERK: Extracellular signal-regulated kinase
EV: Extracellular vesicle
FN: Fibronectin
GI: Genomic instability
GPC-1: Glypican-1
GLUT: Glucose transporter
HER2: Human epidermal growth factor receptor 2
HIF-1α: Hypoxia-inducible factor-1 alpha
HUVEC: Human umbilical vein endothelial cells
IBD: Inflammatory Bowel Disease
IL: Interleukin
ISEV: International Society for Extracellular Vesicles
lncRNA: Long noncoding Ribonucleic acid
LC–MS/MS: Liquid chromatography tandem mass spectrometry
MAPK: Mitogen activated protein kinase
MALAT1: Metastasis Associated Lung Adenocarcinoma transcript 1
MISEV: Minimal information for studies of extracellular vesicles
MMP: Matrix metalloproteinase
M/LEV: Medium/large extracellular vesicle
MRI: Magnetic resonance imaging
MSC: Mesenchymal stem cell
MYC: Myelocytomatosis
NK: Natural Killer
NGS: Next generation sequencing
NSCLC: Non small cell lung cancer
PD-L1: Programmed death-ligand 1
P-gp: P-glycoprotein
PR: Progesterone receptor
PTX: Paclitaxel
PTGFRN: Prostaglandin F2 receptor negative regulator
PSMA: Prostate-specific membrane antigen
SCC: Squamous cell carcinoma
TAM: Tumor-associated macrophage
TEx: Tumor-derived exosomes
TGF-β: Transforming growth factor-beta
TNBC: Triple-negative breast cancer
TME: Tumor microenvironment
UV: Ultraviolet
USMB: Ultrasound stimulated microbubbles
VEGF: Vascular Endothelial Growth Factor
WJ-MSC: Wharton's Jelly MSC
ZO-1: Zona occludens-1
